# Association analysis of weight-adjusted waist index with hypertension and its subtypes

**DOI:** 10.3389/fpubh.2026.1853213

**Published:** 2026-06-26

**Authors:** Lincong Wu, Yixin Ouyang, Yangxue Li, Yang Lu, Changgui Kou, Bin Liu, Junduo Wu

**Affiliations:** 1 Department of Cardiology, Second Hospital of Jilin University, Changchun, Jilin, China; 2 Department of Epidemiology and Biostatistics, School of Public Health, Jilin University, Changchun, Jilin, China

**Keywords:** blood pressure, China, hypertension, mediation effect, weight-adjusted waist index

## Abstract

**Background:**

Hypertension is a leading global risk factor for cardiovascular diseases, with notable regional differences in China. The weight-adjusted waist index (WWI), a novel indicator of central obesity, shows potential in assessing health risks but its link to hypertension subtypes, especially in northeastern China, remains underexplored.

**Methods:**

Using a stratified multistage random sampling approach, we enrolled 6,974 permanent residents aged ≥18 years from six cities in Jilin Province (2021–2022). Multivariate logistic regression examined associations between WWI quartiles and hypertension subtypes. Mediation analysis assessed the potential mediating effects of high-density lipoprotein cholesterol (HDL-C) and low-density lipoprotein cholesterol (LDL-C).

**Results:**

After comprehensive adjustment, the highest WWI quartile (Q4) demonstrated significant associations with overall hypertension risk (adjusted OR = 1.974, 95% CI: 1.611–2.420). Subtype analysis revealed maximum effect sizes for systolic-diastolic hypertension (OR = 2.448, 95% CI: 1.827–3.281), whereas no significant association was observed with isolated diastolic hypertension. Mediation analysis indicated that HDL-C and LDL-C partially mediated the relationship, with mediated proportions of 2.35 and 3.92%. Notably, HDL-C fully mediated IDH (18.2%). ROC analysis showed that WWI had comparable predictive performance to BMI and WC for ISH (*p* > 0.05).

**Conclusion:**

WWI is positively associated with hypertension, particularly SDH, in Northeast Chinese adults. These findings highlight the importance of targeting central obesity in hypertension management. Prospective cohort studies are needed to confirm the temporal relationship and evaluate the clinical utility of WWI.

## 1 Background

Hypertension is a major global risk factor for cardiovascular diseases, such as ischemic heart disease and stroke, and also significantly contributes to the burden of chronic kidney disease (1). Hypertension ranks third among the six leading risk factors that contribute to the global disease burden, following underweight and unsafe sexual behavior (2). From 1990 to 2019, the number of hypertensive individuals aged 30–79 years doubled, from 331 million females and 317 million males to 626 million females and 652 million males in China (3). The insidious nature of hypertension, characterized by the frequent absence of conspicuous symptoms, frequently delays clinical detection and leaves a substantial proportion of affected individuals undiagnosed until severe complications manifest (4, 5). Hypertension is classified into three subtypes based on systolic and diastolic blood pressure levels: isolated systolic hypertension (ISH), isolated diastolic hypertension (IDH), and combined systolic-diastolic hypertension (SDH) (6). Among these, SDH is the most common subtype, followed by ISH and IDH (7). The overall prevalence of hypertension and the proportion of ISH increase linearly with age. By the age of 60, about two-thirds of hypertensive patients have ISH, and by age 75, approximately three-quarters of hypertensive individuals have ISH (8). According to the 2018 national representative survey, the overall prevalence of hypertension in China is approximately 38.1%, with a standardized prevalence of 24.7%. An estimated 274 million individuals aged 18–69 years in China are suffering from hypertension (9). Furthermore, the prevalence of SDH, IDH, and ISH is higher in northern China compared to southern regions (10). Specifically, the northeastern regions of China exhibit a significantly higher prevalence of hypertension when compared to the southern regions, highlighting pronounced regional variations in health profiles (11).

Overweight and obesity are associated with an increased risk of all-cause mortality and significant risk factors for hypertension (12). The weight-adjusted waist index (WWI), an emerging indicator of obesity, is derived by normalizing waist circumference relative to body weight. It primarily reflects central obesity and is not influenced by body weight itself. Unlike Body Mass Index (BMI) or waist circumference (WC), WWI has a high correlation with age, suggesting that it may be relevant to age-related changes in body composition (13).

Several studies have explored the relationship between WWI and hypertension. In the NHANES study, adults in the highest quartile of WWI had a significantly higher prevalence of hypertension compared to those in the lowest quartile (14, 15). In addition, a rural cohort study in Henan province, China, found that individuals in the highest quartile of WWI had a significantly increased risk of hypertension (16). Meanwhile, the CHARLS cohort study, which covered a nationwide sample, revealed a non-linear relationship between WWI and hypertension: when WWI was less than 11.08, it was negatively correlated with hypertension prevalence, while above this threshold, WWI was positively correlated with hypertension (17). However, most current studies focus on regions in the United States of America or specific areas in China, such as southern or rural regions, with limited targeted analyses for northeastern China. Jilin Province, located in northeastern China, presents unique regional characteristics including cold climate conditions and dietary patterns which have high salt and fat intake. These factors may influence obesity distribution patterns, thereby altering epidemiological features of hypertension and its subtypes. Moreover, the majority of studies emphasize the overall prevalence of hypertension, with insufficient investigation into the potential relationship between WWI and specific hypertension subtypes.

Furthermore, recent studies have indicated that WWI is not only linked to hypertension but also correlates with various other metabolic variables, including high-density lipoprotein cholesterol (HDL-C) and low-density lipoprotein cholesterol (LDL-C) (18). These findings suggest that WWI may serve as a valuable marker for assessing overall metabolic health, beyond its association with hypertension alone. Therefore, this study, using a cross-sectional design, is the first to analyze the association between WWI and hypertension, along with its subtypes, in a population from six cities in Jilin Province. This will provide new regional evidence for epidemiological research on hypertension in Jilin.

## 2 Methods

### 2.1 Study population

This study used data from the Jilin province site of the China Hypertension Survey, a national cardiovascular disease survey led by Fuwai Hospital, Chinese Academy of Medical Sciences. The authors participated in this survey as co-investigators at the Jilin site, contributing to data collection. The survey employed a stratified multistage random sampling design and was conducted from August 2021 to 2022. The four-stage sampling procedure was as follows:

Stage 1: Stratification and sampling of counties/districts. The study area was stratified into rural and urban regions. Within each stratum, four counties/districts were selected using Probability Proportional to Size (PPS) sampling.
Stage 2: Random selection of townships/streets within the chosen counties/districts. Two townships/streets were randomly chosen from each selected county/district using Simple Random Sampling (SRS).
Stage 3: Further sampling of neighborhood committees/village committees within the selected townships/streets. Three neighborhood committees/village committees were randomly selected from each township/street using SRS. If the projected sample size of adults aged 18 years and above in the selected villages was insufficient to meet the minimum requirement for a specific age group, adjustments were made based on geographical proximity, merging smaller villages or replacing them with larger neighboring villages.
Stage 4: Household visits to recruit eligible individuals. Within the selected neighborhood/village committees, individuals were stratified by gender and age into 14 groups (18–24, 25–34, 35–44, 45–54, 55–64, 65–74, ≥75 years) and randomly selected using SRS. Eligibility criteria included being a resident aged 18 years or older who had lived in the area for at least 6 months within the past year.

**Figure 1:**
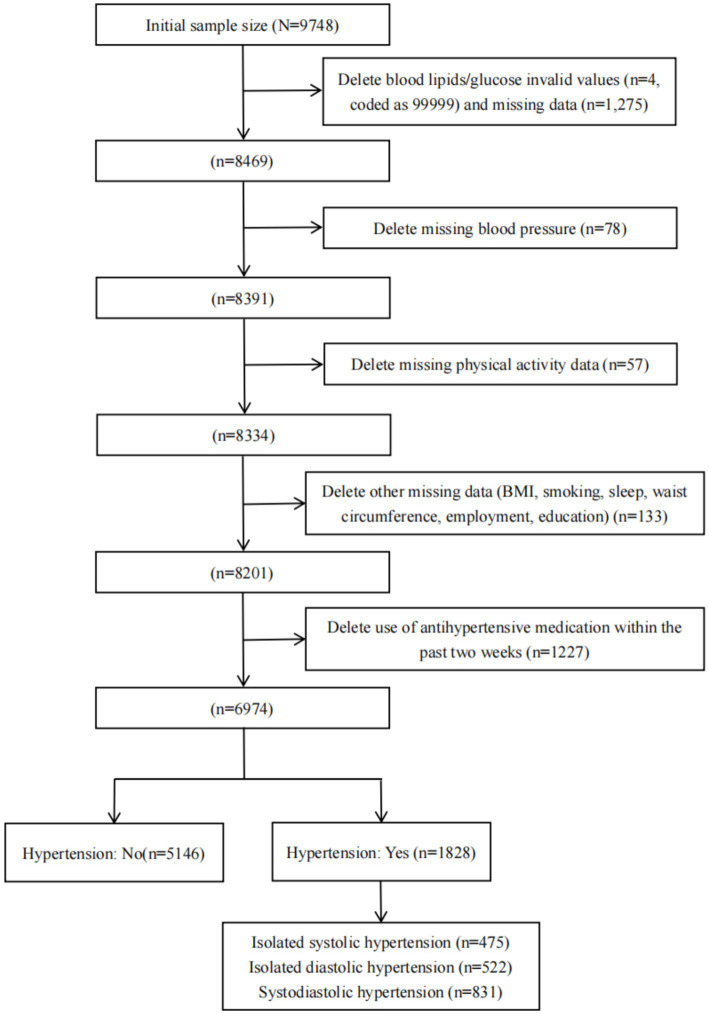
Inclusion and exclusion criteria flowchart.

In quality control process, participants were required to have complete demographic, behavioral, and metabolic data, as well as complete blood pressure records. Cases with incomplete data were excluded. A total of 9,748 individuals were surveyed from August 2021 to December 2022, and 6,974 eligible participants were included in the final analysis (Figure 1).

Written informed consent was obtained from all participants prior to data collection. The study protocol was approved by the Ethics Committee of Fuwai Hospital (Approval No. 2020–1,360, dated July 30, 2020, valid for 2 years). All analyses in this study were performed using de-identified data.

### 2.2 Definition and classification of hypertension

This study adheres to the Chinese Hypertension League (CHL) guidelines for the diagnosis and classification of blood pressure (19). Normal blood pressure was defined as systolic pressure <140 mmHg and diastolic pressure <90 mmHg in the absence of antihypertensive medication use. Hypertension was defined according to the standard clinical criteria: systolic pressure ≥140 mmHg and/or diastolic pressure ≥90 mmHg, or current use of antihypertensive medication within the past 2 weeks.

Blood pressure was measured by trained medical professionals using a standardized electronic sphygmomanometer (device model Omron HBP 1120 U) with an accuracy of 1 mmHg. All devices were calibrated by the manufacturer and certified by the national quality inspection department. Participants were required to avoid vigorous exercise, eating, and drinking (especially caffeinated beverages such as tea and coffee) within 1 h before measurement and to abstain from smoking within 30 min. After resting quietly for 5 min in a sitting position, three blood pressure readings were taken from the right upper arm (supported at heart level), with at least a one-minute interval between measurements. The three readings were recorded, and if the difference between any two systolic or diastolic pressure readings exceeded 10 mmHg, the measurements were repeated after a brief rest until the criteria were met. The average of the three acceptable readings was used as the final blood pressure value. Hypertension was further classified into three subtypes:

Isolated systolic hypertension: SBP ≥ 140 mmHg and DBP < 90 mmHg.
Isolated diastolic hypertension: DBP ≥ 90 mmHg and SBP < 140 mmHg.
Systolic-diastolic hypertension: SBP ≥ 140 mmHg and DBP ≥ 90 mmHg (20, 21).

### 2.3 Definition of weight-adjusted waist index

WWI was calculated as the WC (cm) divided by the square root of body weight (kg), reflecting the proportional relationship between an individual’s waist circumference and body weight (22). Participants were divided into four groups based on the quartiles of WWI: Q1 (≤25th percentile), Q2 (25th percentile < value ≤ 50th percentile), Q3 (50th percentile < value ≤ 75th percentile), and Q4 (>75th percentile).

During measurement, participants removed heavy clothing and items from their pockets. Weight was measured to an accuracy of 0.1 kg. WC was measured at the end of expiration, with readings accurate to 0.1 cm. Two measurements were taken, and if the difference exceeded 0.5 cm, the measurement was repeated.

### 2.4 Covariates

To control for potential confounding factors, the study adjusted for sociodemographic characteristics, behavioral habits, and metabolic indicators as follows:

Sociodemographic factors: Gender (male/female), age (continuous, years), ethnicity (Han/other ethnicities), employment, education, residence and annual income.
Behavioral habits: Smoking status (current smoker: yes/no), alcohol consumption (current drinker: yes/no), physical activity (daily activity time, minutes), and sleep time.
Metabolic indicators: Fasting blood glucose (GLU, mmol/L), HDL-C (mmol/L), and LDL-C (mmol/L).
Family history: Presence of cardiovascular disease in family history (yes/no).

### 2.5 Statistical analysis

Descriptive statistics were used to summarize the sociodemographic characteristics of the study population. Categorical variables were expressed as frequencies and percentages, while continuous variables were presented as medians and interquartile ranges (IQR). Differences between groups were analyzed using chi-square tests for categorical variables or Mann–Whitney U tests for continuous variables.

The main effect analysis employed multivariable logistic regression models to assess the association between WWI and hypertension. Three models were constructed:

Model 1 is an unadjusted model that includes only the WWI quartiles. Model 2 adjusted for gender, age, ethnicity, employment, education, residence and annual income in addition to the WWI quartiles. Model 3 further adjusted for smoking status, alcohol consumption, physical activity, GLU, HDL-C, LDL-C, and family history of cardiovascular diseases, in addition to the variables in Model 2. In addition to quartile analysis, WWI was also analyzed as a continuous variable (per 1-unit increase) to assess its linear association with hypertension and its subtypes.

Based on Model 3, restricted cubic splines (RCS) with five knots (the 5th, 27.5th, 50th, 72.5th and 95th percentiles) were fitted for WWI. A likelihood ratio test comparing the linear and spline models was used to test for non-linearity. The threshold (inflection point) was defined as the WWI value at which the lower bound of the OR’s 95% CI first exceeded 1.

In addition to the main effect analysis, mediation analyses were performed separately for HTN and its three subtypes (ISH, IDH, and SDH), using HDL-C and LDL-C as mediators. The dataset was randomly split into training (70%) and test (30%) sets. Models using WWI, BMI, or WC were fitted on the training set, and area under the curve (AUC)s with 95% CIs were calculated on the test set, and Delong’s test was used to compare the AUCs. Abdominal obesity precedes dyslipidemia and hypertension in the natural history of metabolic syndrome, as supported by prospective cohort studies (23, 24). However, given the cross-sectional design, the assumed temporal ordering (WWI → lipids → hypertension) cannot be definitively established, and reverse causation is equally plausible. Thus, our mediation findings are exploratory.

In the sensitivity analyses, we redefined hypertension solely by measured blood pressure (≥140/90 mmHg). BMI was not included in the primary analysis because WWI and BMI show substantial collinearity [GVIF = 7.056 in Wang et al. (25)], which could cause unstable estimates or reversed effect direction. However, to assess the independent contribution of WWI beyond BMI, we added BMI to Model 3 as a sensitivity analysis. Additionally, to examine whether socioeconomic status modified the association between WWI and hypertension, we added the product term “WWI* annual income” to Model 3. The same statistical methods used in the main effect analysis were applied to assess the robustness of our findings.

*A priori* power calculation was not performed. Post-hoc power calculations were conducted using the “pwr” package in R based on the observed ORs from Model 3, assuming a two-sided *α* level of 0.05.

All analyses were performed using IBM SPSS Statistics (SPSS Inc., Chicago, IL, USA) (Version 24.0) and R (version 4.3.3) with two-sided tests and a significance level of *p* < 0.05.

## 3 Results

The study included 6,974 participants. The mean WWI was 10.29, and the prevalence rates were 26.21% for HTN, 6.81% for ISH, 7.48% for IDH, and 11.92% for SDH. The distribution of baseline characteristics could be seen in Table 1. The median age of the study was 42 years (IQR: 33.00 to 53.00 years), with a slightly higher proportion of females (51.09%) compared to males (48.91%). In terms of educational attainment, 31.40% of participants had a diploma or higher education, while 45.20% had an educational level of junior high school or below. Regarding urban–rural distribution, rural residents accounted for 56.81%, slightly outnumbering the urban residents. Behavioral characteristics indicated that 29.04% were smokers and 34.73% were alcohol consumers.

**Table 1:** Sociodemographic characteristics of the study population (*n* = 6,974).

Variable	Category	*n* (%)
Age (*P*_50_, *P*_25_, *P*_75_)		42.00 (31.00, 53.00)
Gender	Male	3,411 (48.91)
	Female	3,563 (51.09)
Annual income	<10,000 CNY	1,142 (16.38)
	10,000–49,999 CNY	4,861 (69.70)
	≥50,000 CNY	971 (13.92)
Education level	Illiterate	176 (2.52)
	Primary school	960 (13.77)
	Junior high school	2016 (28.91)
	High school/technical school	1,632 (23.40)
	College/higher	2,190 (31.40)
Employment status	Employed	3,435 (49.25)
	Students	661 (9.48)
	Retired	710 (10.18)
	Other	2,168 (31.09)
Ethnicity	Han	6,065 (86.97)
	Others	909 (13.03)
Residence	Urban	3,012 (43.19)
	Rural	3,962 (56.81)
Marital status	Unmarried	1,521 (21.81)
	Married/remarried/cohabiting	4,932 (70.72)
	Divorced	265 (3.80)
	Widowed	256 (3.67)
Smoking status	No	4,949 (70.96)
	Yes	2025 (29.04)
Alcohol status	No	4,552 (65.27)
	Yes	2,422 (34.73)

The prevalence of hypertension varied significantly across different sociodemographic characteristics, as detailed in Table S1. The incidence of hypertension was notably higher among males (35.77%) compared to females (17.06%, *p* < 0.001). Ethnicity also played a role, with the Han exhibiting a higher prevalence rate of 27.37% compared to other ethnic groups at 18.48% (*p* < 0.001). Urban–rural differences were evident as well: rural residents had a hypertension prevalence of 27.39%, surpassing the 24.67% observed in urban populations (*p* < 0.05). These disparities suggest that various social and environmental factors may significantly influence the development of hypertension.

In the regression analysis examining the association between WWI and hypertension (Table 2), a significant positive correlation was observed. In the unadjusted model (Model 1), participants in Q4 had a substantially increased risk of hypertension compared to those in Q1 (OR = 4.247, 95% *CI*: 3.565–5.060, *p* < 0.001). After adjusting for sociodemographic factors (Model 2), the risk estimate for Q4 decreased but remained significant (OR = 2.114, 95% *CI*: 1.732–2.580). Further adjustment in Model 3 for behavioral and metabolic factors still demonstrated a strong association (OR = 1.974, 95% *CI*: 1.611–2.420, *p* < 0.001), indicating that WWI is a risk factor for hypertension. As a continuous variable, each 1-unit increase in WWI was associated with a 32% higher odds of hypertension (OR = 1.320, 95% CI: 1.218–1.431, *p* < 0.001). For hypertension subtypes, WWI showed the strongest association with SDH (Q4: OR = 2.448, 95% CI: 1.827–3.281, *p* < 0.001), followed by ISH (Q4: OR = 2.375, 95% CI: 1.622–3.477, *p* < 0.001), while no significant association was observed for IDH (Q4: OR = 1.253, 95% CI: 0.907–1.730, *p* = 0.172). The continuous analysis yielded consistent results (ISH: OR = 1.441, *p* < 0.001; SDH: OR = 1.353, *p* < 0.001; IDH: OR = 1.136, *p* = 0.060).

**Table 2:** Association between WWI quartiles and HTN, ISH, IDH, and SDH.

WWI	Model 1^a^		Model 2^b^		Model 3^c^	
	OR (95%CI)	*P*	OR (95%CI)	*P*	OR (95%CI)	*P*
HTN						
WWI	1.757 (1.646,2.876)	<0.001	1.351 (1.248,1.462)	<0.001	1.320 (1.218,1.431)	<0.001
Q1	1.000 (Ref)	–	1.000 (Ref)	–	1.000 (Ref)	–
Q2	2.225 (1.853,2.673)	<0.001	1.487 (1.223,1.808)	<0.001	1.442 (1.183,1.757)	<0.001
Q3	3.820 (3.204,4.556)	<0.001	2.158 (1.782,2.612)	<0.001	2.038 (1.678,2.475)	<0.001
Q4	4.247 (3.565,5.060)	<0.001	2.114 (1.732,2.580)	<0.001	1.974 (1.611,2.420)	<0.001
*P* for trend		<0.001		<0.001		<0.001
ISH						
WWI	2.476 (2.214,2.769)	<0.001	1.454 (1.276,1.658)	<0.001	1.441 (1.263,1.644)	<0.001
Q1	1.000 (Ref)	–	1.000 (Ref)	–	1.000 (Ref)	–
Q2	1.901 (1.299,2.784)	0.001	1.382 (0.922,2.072)	0.117	1.377 (0.915,2.071)	0.125
Q3	3.759 (2.643,5.347)	<0.001	1.994 (1.358,2.927)	<0.001	1.963 (1.332,2.894)	0.001
Q4	7.362 (5.287,10.253)	<0.001	2.453 (1.683,3.578)	<0.001	2.375 (1.622,3.477)	<0.001
*P* for trend		<0.001		<0.001		<0.001
IDH						
WWI	1.298 (1.166, 1.445)	<0.001	1.181 (1.037, 1.346)	0.012	1.136 (0.995, 1.298)	0.060
Q1	1.000 (Ref)	–	1.000 (Ref)	–	1.000 (Ref)	–
Q2	1.818 (1.377, 2.401)	<0.001	1.322 (0.991, 1.765)	0.058	1.258 (0.940, 1.682)	0.123
Q3	2.836 (2.174, 3.701)	<0.001	1.953 (1.472, 2.591)	<0.001	1.802 (1.353, 2.398)	<0.001
Q4	1.782 (1.333, 2.383)	<0.001	1.372 (0.998, 1.885)	0.051	1.253 (0.907, 1.730)	0.172
*P* for trend		0.015		0.134		0.374
SDH						
WWI	1.764 (1.615, 1.926)	<0.001	1.377 (1.236, 1.534)	<0.001	1.353 (1.212, 1.512)	<0.001
Q1	1.000 (Ref)	–	1.000 (Ref)	–	1.000 (Ref)	–
Q2	2.888 (2.189, 3.811)	<0.001	1.779 (1.335, 2.371)	<0.001	1.725 (1.291, 2.305)	<0.001
Q3	4.998 (3.831, 6.520)	<0.001	2.606 (1.972, 3.443)	<0.001	2.473 (1.865, 3.280)	<0.001
Q4	5.279 (4.047, 6.886)	<0.001	1.779 (1.335, 2.371)	<0.001	2.448 (1.827, 3.281)	<0.001
*P* for trend		<0.001		<0.001		<0.001

WWI, Weight-Adjusted Waist Index; HTN, hypertension; ISH, isolated systolic HTN; IDH, isolated diastolic HTN; SDH, systolic-diastolic HTN; CI, confidence interval; OR, odds ratio. Model 1^a^: Unadjusted. Model 2^b^: Adjusted for gender, age, ethnicity, employment, education, residence and annual income. Model 3^c^: Further adjusted for physical activity, sleep time, smoking, alcohol consumption, GLU, HDL-C, LDL-C, and family history of cardiovascular disease.

**Figure 2:**
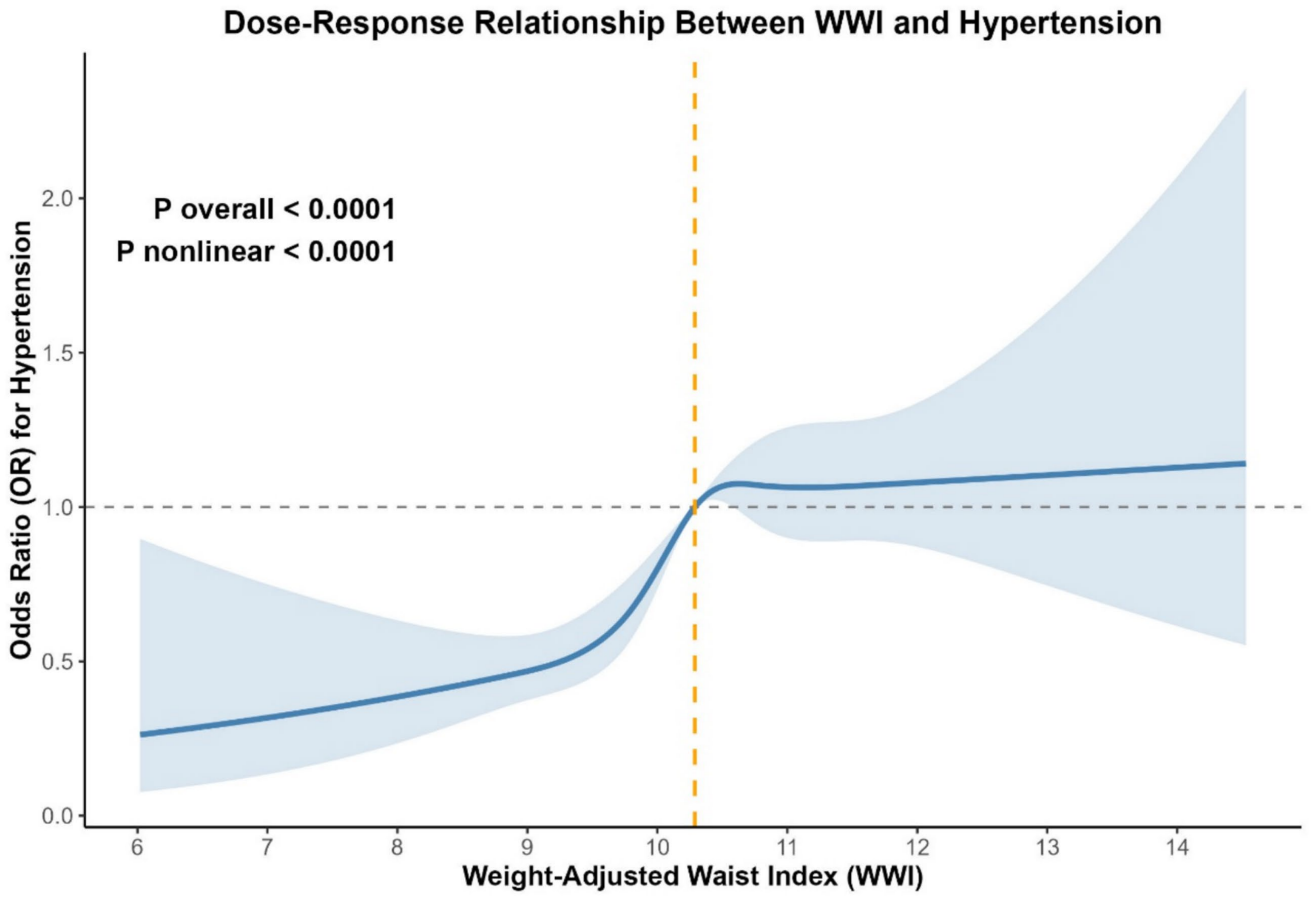
Dose–response relationship between WWI and hypertension.

RCS analysis with five knots (located at the 5th, 27.5th, 50th, 72.5th and 95th percentiles of the WWI distribution: 8.85, 9.78, 10.29, 10.78, and 11.69) was performed. The results revealed a significant non-linear relationship between WWI and hypertension (likelihood ratio test: χ^2^ = 24.20, df = 3, *p* = 2.27 × 10^−5^; Figure 2). The 95% CI lower bound first crossed 1 at WWI = 10.301, indicating the point at which the OR became statistically significant. The OR then continued to increase until approximately 10.6, after which the curve plateaued. At WWI levels above the 95th percentile (> 11.69), the confidence intervals widened substantially, indicating reduced statistical precision due to sparse data in the upper tail of the exposure distribution (Supplementary Table S2).

**Figure 3:**
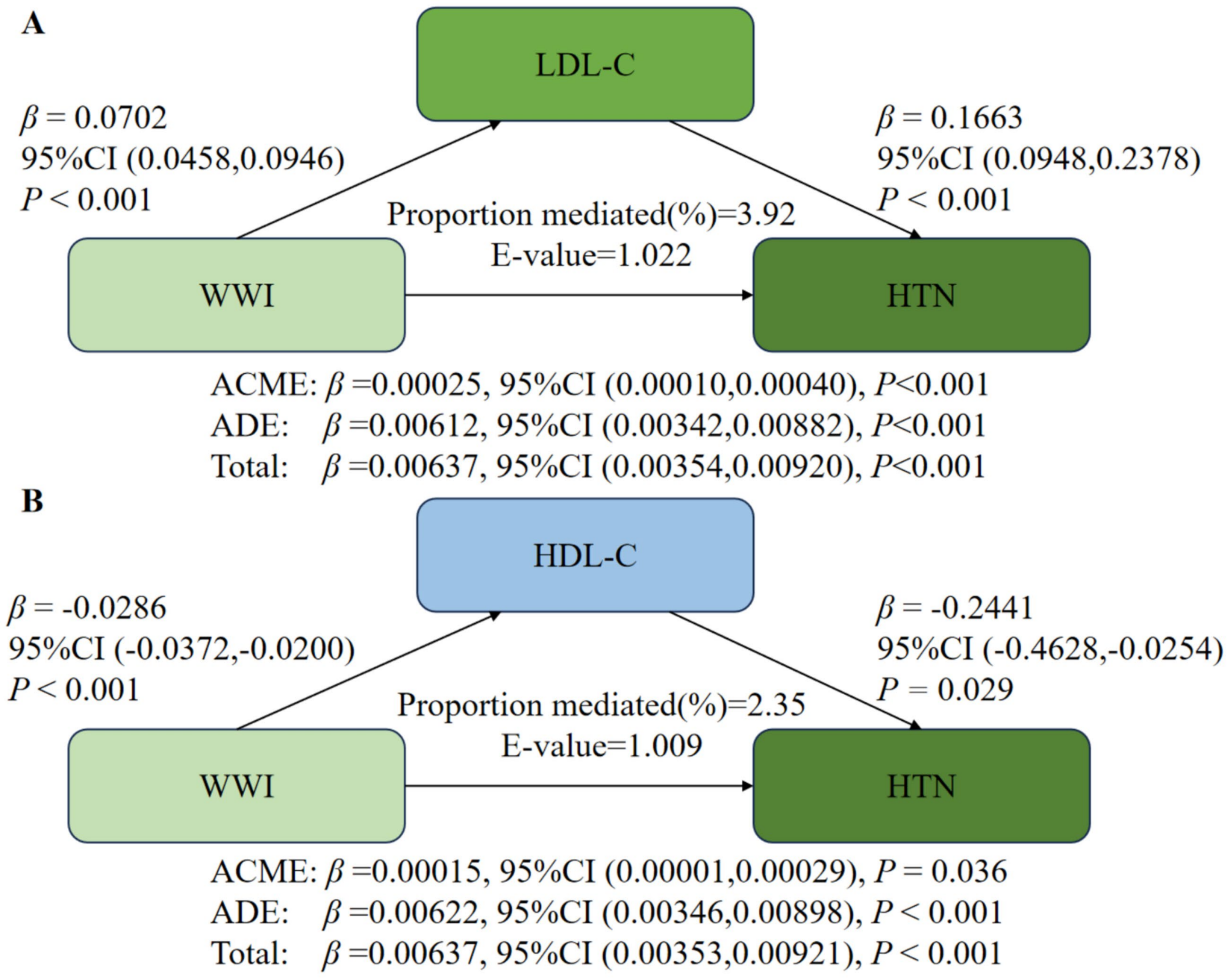
Mediation analysis of HDL-C and LDL-C in the WWI-hypertension association.

Mediation analysis revealed that both HDL-C and LDL-C significantly mediated the relationship between WWI and hypertension. Specifically, LDL-C accounted for 3.92% of the effect of WWI on hypertension, while HDL-C mediated 2.35% of this relationship (Figure 3). Notably, HDL-C fully mediated IDH (18.2%) for the total effect. However, E-value analysis indicated high sensitivity to unmeasured confounding, with E-values of 1.009 for HDL-C and 1.022 for LDL-C (Supplementary Table S2).

In the test set, WWI achieved an AUC of 0.764 (95% CI: 0.744–0.787) for overall hypertension, compared to 0.789 (95% CI: 0.777–0.817) for BMI and 0.783 (95% CI: 0.771–0.812) for WC (both *p* < 0.001 vs. WWI). Similar patterns were observed across hypertension subtypes. For ISH, WWI showed comparable performance (AUC = 0.772) to BMI (0.783) and WC (0.781) (both *p* > 0.05). For IDH and SDH, BMI and WC had significantly higher AUCs than WWI (both *p* < 0.05) (Supplementary Figure S1).

Sensitivity analyses (Supplementary Table S3) further corroborated these findings. Specifically, in the SDH subgroup, participants in Q4 exhibited a significantly elevated risk (OR = 2.156, 95% *CI*: 1.702–2.730, *p* < 0.001). However, no significant association was found in the IDH group (OR = 1.304, 95% CI: 0.965–1.761, *p* = 0.084), suggesting that WWI may have a more pronounced effect on hypertension subtypes associated with systolic blood pressure elevation. After adjusting for BMI, WWI remained significantly associated with HTN, ISH, and SDH, albeit with attenuated effect sizes, supporting the robustness of our findings. VIF analysis confirmed no collinearity between WWI and BMI, indicating that WWI provides independent predictive value beyond BMI (Supplementary Tables S4–S6).

The interaction term “WWI*annual income” was not statistically significant (*P* for interaction = 0.460), indicating that the association between WWI and hypertension did not differ significantly across income levels.

Post-hoc power analysis showed that the study had 100% power for HTN (OR = 1.320, *n* = 1,828), 99.5% power for ISH (OR = 1.441, *n* = 475), 34.8% power for IDH (OR = 1.136, *n* = 522), and 99.9% power for SDH (OR = 1.353, *n* = 831).

## 4 Discussion

This study, based on a cross-sectional survey of populations in six cities in Jilin Province, is the first to investigate the association between the WWI and hypertension and its subtypes. Our findings reveal a significant positive correlation between WWI and hypertension prevalence, particularly in SDH. Specifically, individuals in Q4 had a significantly higher risk of hypertension than those in Q1 (OR = 1.974, 95% CI: 1.611–2.420). Mediation analysis further showed that HDL-C and LDL-C partially mediated this association, with mediated proportions of 2.35 and 3.92%, respectively. Notably, HDL-C fully mediated IDH (18.2%), while LDL-C partially mediated SDH (4.84%).

These mediating roles are biologically well-founded. Abdominal obesity, as captured by WWI, promotes insulin resistance and chronic low-grade inflammation, leading to dyslipidemia characterized by reduced HDL-C and elevated LDL-C (26). HDL-C exerts antihypertensive effects through multiple pathways: it enhances reverse cholesterol transport, improves endothelial function by promoting nitric oxide bioavailability, and possesses antioxidant and anti-inflammatory properties that protect against vascular damage (27, 28). Conversely, LDL-C contributes to hypertension by inducing endothelial dysfunction, increasing arterial stiffness, and activating the renin-angiotensin-aldosterone system (RAAS) (29). Oxidized LDL-C further promotes vascular inflammation and smooth muscle cell proliferation, leading to increased peripheral vascular resistance (30). These pathways explain the opposing mediation directions observed in our study: HDL-C negatively mediates (protective effect), while LDL-C positively mediates (risk-enhancing effect).

The RCS inflection point in our study (10.301) was lower than that reported in CHARLS (11.08) (17). This difference likely reflects age distribution disparities. CHARLS included adults ≥45 years, where age itself confers high risk, requiring higher WWI to manifest an effect. Our study included individuals ≥18 years, who have better metabolic flexibility and greater sensitivity to WWI. Thus, the threshold difference is biologically plausible, not contradictory. Notably, beyond the 95th percentile of WWI (> 11.69), confidence intervals widened substantially and the risk plateaued, indicating reduced precision in the upper tail; these estimates should be interpreted with caution.

Our findings are consistent with multiple studies both domestically and internationally, confirming a significant positive correlation between WWI and hypertension prevalence. For instance, an analysis of 37,299 participants from the NHANES database (2005–2018) revealed that the hypertension prevalence in the highest WWI quartile was 2.27 times that of the lowest quartile (OR = 2.27, 95% CI: 1.97–2.61, *p* < 0.001) (15). Similarly, Sun et al. reported a stronger association, with an OR of 2.94 (95% CI: 2.65–3.27, *p* < 0.001) (31). The maintained significance after excluding individuals using antihypertensive medications further validates the robustness of WWI as a risk factor. However, the non-significant association between WWI and isolated diastolic hypertension may be related to the multifactorial nature of diastolic pressure regulation, such as vascular elasticity and peripheral resistance, which requires further investigation (32, 33).

Traditional obesity metrics like BMI and WC have long been used in clinical practice, but their limitations are increasingly recognized. BMI cannot distinguish between muscle and fat mass (34) and WC correlates strongly with BMI, limiting its independence (35). Emerging evidence has shown that WWI may serve as a useful indicator for various obesity-related comorbidities, including chronic kidney disease (36), cardiovascular diseases (37), diabetic nephropathy (38), hyperuricemia (39), depressive symptoms (40), and non-alcoholic fatty liver disease and liver fibrosis (41). In our study, ROC analysis revealed that BMI and WC exhibited stronger discriminatory power for hypertension than WWI (AUC: 0.789 and 0.783 vs. 0.764). However, for ISH, WWI demonstrated comparable predictive performance to BMI and WC, with no statistically significant differences in AUC values (*p* > 0.05). This finding is of particular interest because ISH is primarily driven by arterial stiffness rather than metabolic abnormalities (42). As populations age, aortic stiffening leads to increased pulse pressure and isolated systolic hypertension (43). Traditional obesity indicators like BMI may not fully capture this vascular pathology. Therefore, WWI, which reflects weight-independent central obesity, may serve as a valuable tool for assessing ISH risk, a subtype closely linked to arterial stiffness.

The association between WWI and hypertension may involve multiple physiological mechanisms. Abdominal obesity may lead to blood pressure elevation through its impact on renal function, causing sodium retention and subsequent expansion of plasma volume, which becomes a potential trigger for blood pressure increase (44, 45). Visceral fat accumulation induces endocrine dysregulation, particularly insulin resistance, while triggering pro-inflammatory cytokine release (e.g., interleukin-6: IL-6, tumor necrosis factor-*α*: TNF-α, monocyte chemoattractant protein: MCP-1) that promotes vascular endothelial dysfunction (46, 47). In addition, the overactivation of the sympathetic nervous system is also considered an important mechanism in the development of hypertension, as it can lead to increased heart rate and vasoconstriction, further increasing the risk of hypertension (48).

The strengths of this study lie in its pioneering exploration of the association between WWI and hypertension subtypes, providing new regional evidence for the epidemiology of hypertension in Jilin Province. Moreover, the use of a stratified multistage random sampling method ensures the representativeness of the sample.

However, several limitations should be noted. First, as a cross-sectional study, we cannot establish causality between WWI and hypertension but can only reveal their association. Second, our analysis did not account for all potential confounders, such as dietary factors, sodium intake, or renal function indicators (eGFR, urinary albumin). Although sleep duration was included as a covariate in Model 3, sleep quality was not assessed. Third, antihypertensive medication was included in the hypertension definition (SBP/DBP ≥ 140/90 or current medication use), not excluded. Thus, medication adherence and class were not separately analyzed. Fourth, ethnicity was dichotomized as Han versus other due to limited sample sizes of minority groups (e.g., Korean, Manchu, Mongolian), which may obscure ethnic differences in hypertension risk. Fifth, the regional scope (Jilin Province) may limit the generalizability. Sixth, the cross-sectional design precludes causal inference for the mediation pathways, and reverse causation cannot be ruled out. Finally, post-hoc power analysis indicated that the null finding for IDH may reflect limited statistical power rather than a true absence of association. Larger prospective studies are needed to confirm the relationship between WWI and IDH.

Future research could focus on clarifying the potential mechanisms underlying the relationship between WWI and hypertension subtypes. Prospective cohort studies are needed to establish the temporal relationship and predictive value. Additionally, investigations into regional factors such as cold climate and high-sodium diets may provide further insights into the role of environmental factors in modulating the association between WWI and hypertension.

## 5 Conclusion

In this cross-sectional study, WWI was positively associated with hypertension, particularly SDH, among adults in Northeast China. The association between central obesity and blood pressure elevation was primarily driven by systolic pressure. These findings provide a rationale for developing targeted obesity management strategies aimed at systolic blood pressure control.

## Data Availability

The datasets generated and/or analyzed during the current study are not publicly available due to ethical restrictions. However, they are available from the corresponding author on reasonable request. Requests to access the datasets should be directed to JW, wujd@jlu.edu.cn.

## Glossary

WWI: Weight-adjusted Waist Index
HDL-C: High-density lipoprotein cholesterol
LDL-C: Low-density lipoprotein cholesterol
HTN: Hypertension
ISH: Isolated systolic hypertension
IDH: Isolated diastolic hypertension
SDH: Systolic-diastolic hypertension
PPS: Probability Proportional to Size
SRS: Simple Random Sampling
CHL: Chinese Hypertension League
GLU: Fasting blood glucose
IQR: Interquartile ranges
BMI: Body Mass Index
WC: Waist Circumference
RCS: Restricted Cubic Splines
AUC: Area Under the Curve
Q1: ≤25th percentile
Q2: 25th percentile < value ≤ 50th percentile
Q3: 50th percentile < value ≤ 75th percentile
Q4: >75th percentile
RAAS: Renin-Angiotensin-Aldosterone System
