# Supplementary material for: Association analysis of weight-adjusted waist index with hypertension and its subtypes
Source: Front Public Health. 2026 Jun 26;14:1853213. doi: 10.3389/fpubh.2026.1853213 (PMC13350440; doi:10.3389/fpubh.2026.1853213)
Supplement: Supplementary file 1 [file Data_Sheet_1.docx]

**Association Analysis of Weight-Adjusted Waist Index (WWI) with Hypertension and Its Subtypes**

Supplementary Data:

1. Table S1. Hypertension Prevalence by Sociodemographic Characteristics
2. Table S2. Distribution of WWI and hypertension cases across WWI percentiles
3. Table S3. Mediation analysis results for hypertension and its subtypes
4. Table S4. Sensitivity Analysis: Blood pressure-based definition of hypertension (≥140/90 mmHg) excluding participants on antihypertensive medication
5. Table S5. Sensitivity analysis: Association between WWI quartiles and hypertension subtypes after further adjustment for BMI in Model 3
6. Table S6. Multicollinearity diagnostics for covariates in the primary Model 3
7. Table S7. Multicollinearity diagnostics for covariates in the sensitivity analysis
8. Fig. S1.ROC curves of WWI, BMI, and WC for predicting hypertension and its subtypes.

| Table S1 Hypertension Prevalence by Sociodemographic Characteristics | | | | |
| --- | --- | --- | --- | --- |
| Variable | Category | Prevalence (%) | Χ^2^ | *P* |
|  |  |  |  |  |
| Gender |  |  | 315.157 | <0.001 |
|  | Male | 35.77 |  |  |
|  | Female | 17.06 |  |  |
| Ethnicity |  |  | 32.290 | <0.001 |
|  | Han | 27.37 |  |  |
|  | Others | 18.48 |  |  |
| Residence |  |  | 6.532 | 0.011 |
|  | Urban | 24.67 |  |  |
|  | Rural | 27.39 |  |  |
| Employment Status |  |  | 225.387 | <0.001 |
|  | Employed | 26.17 |  |  |
|  | Students | 6.05 |  |  |
|  | Retired | 41.41 |  |  |
|  | Other | 27.44 |  |  |

| Table S2. Distribution of WWI and hypertension cases across WWI percentiles | | | |
| --- | --- | --- | --- |
| WWI Percentile | WWI threshold | N cumulative | N events cumulative |
| 5th | 8.85 | 349 | 28 |
| 10th | 9.17 | 698 | 59 |
| 25th | 9.72 | 1744 | 206 |
| 50th | 10.29 | 3488 | 606 |
| 75th | 10.84 | 5230 | 1195 |
| 90th | 11.36 | 6276 | 1579 |
| 95th | 11.69 | 6625 | 1697 |
| 99th | 12.43 | 6904 | 1799 |

| Table S3. Mediation analysis results for hypertension and its subtypes | | | | | | |
| --- | --- | --- | --- | --- | --- | --- |
| Outcome | Mediator | Effect type | Risk Difference (95% CI) | *P* | Proportion mediated (%) | E-value |
| ISH | HDL-C | ACME | 0.00001 (-0.00004, 0.00006) | 0.572 | 0.85 | - |
|  |  | ADE | 0.00111 (0.00045, 0.00177) | <0.001 |  |  |
|  |  | Total | 0.00112 (0.00045, 0.00177) | <0.001 |  |  |
|  | LDL-C | ACME | 0.00002 (-0.00001, 0.00005) | 0.152 | 1.82 | - |
|  |  | ADE | 0.00107 (0.00042, 0.00172) | <0.001 |  |  |
|  |  | Total | 0.00109 (0.00042, 0.00176) | <0.001 |  |  |
| IDH | HDL-C | ACME | 0.00051 (0.00012, 0.00091) | <0.001 | 18.20 | 1.016 |
|  |  | ADE | 0.00231 (-0.00455, 0.00917) | 0.224 |  |  |
|  |  | Total | 0.00283 (-0.00341, 0.00907) | 0.140 |  |  |
|  | LDL-C | ACME | 0.00023 (-0.00003, 0.00049) | 0.080 | 8.25 | - |
|  |  | ADE | 0.00257 (-0.00437, 0.00951) | 0.204 |  |  |
|  |  | Total | 0.00280 (-0.00395, 0.00955) | 0.150 |  |  |
| SDH | HDL-C | ACME | 0.000002 (-0.00010, 0.00011) | 0.988 | 0.08 | - |
|  |  | ADE | 0.00278 (0.00129, 0.00427) | <0.001 |  |  |
|  |  | Total | 0.00278 (0.00129, 0.00427) | <0.001 |  |  |
|  | LDL-C | ACME | 0.00014 (0.00004, 0.00024) | <0.001 | 4.84 | 1.008 |
|  |  | ADE | 0.00266 (0.00123, 0.00409) | <0.001 |  |  |
|  |  | Total | 0.00280 (0.00129, 0.00431) | <0.001 |  |  |
| Abbreviations: HTN, hypertension; ISH, isolated systolic hypertension; IDH, isolated diastolic hypertension; SDH, systolic-diastolic hypertension; ACME, average causal mediation effect; ADE, average direct effect; CI, confidence interval. Note: Effects are reported on the risk difference scale. ACME represents the indirect effect.  E-values are reported only for significant mediation effects (*P* < 0.05). | | | | | | |

| Table S4. Sensitivity Analysis: Blood pressure-based definition of hypertension (≥140/90 mmHg) excluding participants on antihypertensive medication | | | | | | |
| --- | --- | --- | --- | --- | --- | --- |
| WWI Quartile | Model 1^a^ | | Model 2^b^ | | Model 3^c^ | |
|  | OR (95%CI) | *P* | OR (95%CI) | *P* | OR (95%CI) | *P* |
| HTN |  |  |  |  |  |  |
| WWI | 1.863 (1.761,1.972) | <0.001 | 1.342 (1.253,1.436) | <0.001 | 1.311 (1.223,1.406) | <0.001 |
| Q1 | 1.000 (Ref) | - | 1.000 (Ref) | - | 1.000 (Ref) | - |
| Q2 | 2.369 (2.032,2.761) | <0.001 | 1.543 (1.307,1.821) | <0.001 | 1.476 (1.248,1.746) | <0.001 |
| Q3 | 4.005 (3.450,4.650) | <0.001 | 2.097 (1.778,2.473) | <0.001 | 1.964 (1.661,2.323) | <0.001 |
| Q4 | 4.651 (4.008,5.397) | <0.001 | 2.023 (1.702,2.405) | <0.001 | 1.891 (1.585,2.257) | <0.001 |
| *P* for trend |  | <0.001 |  | <0.001 |  | <0.001 |
| ISH |  |  |  |  |  |  |
| WWI | 2.231 (2.048,2.430) | <0.001 | 1.385 (1.247,1.539) | <0.001 | 1.371 (1.232,1.525) | <0.001 |
| Q1 | 1.000 (Ref) | - | 1.000 (Ref) | - | 1.000 (Ref) | - |
| Q2 | 1.939 (1.430,2.629) | <0.001 | 1.291 (0.927,1.797) | 0.130 | 1.259 (0.903,1.756) | 0.175 |
| Q3 | 4.386 (3.320,5.794) | <0.001 | 1.973 (1.445,2.695) | <0.001 | 1.904 (1.391,2.608) | <0.001 |
| Q4 | 8.391 (6.443,10.928) | <0.001 | 2.120 (1.557,2.885) | <0.001 | 2.049 (1.500,2.799) | <0.001 |
| *P* for trend |  | <0.001 |  | <0.001 |  | <0.001 |
| IDH |  |  |  |  |  |  |
| WWI | 1.050 (0.959,1.150) | 0.287 | 1.202 (1.067,1.354) | 0.002 | 1.154 (1.022,1.303) | 0.021 |
| Q1 | 1.000 (Ref) | - | 1.000 (Ref) | - | 1.000 (Ref) | - |
| Q2 | 1.979 (1.542,2.540) | <0.001 | 1.431 (1.105,1.854) | 0.007 | 1.346 (1.037,1.747) | 0.026 |
| Q3 | 2.857 (2.240,3.643) | <0.001 | 1.973 (1.521,2.558) | <0.001 | 1.820 (1.399,2.367) | <0.001 |
| Q4 | 1.757 (1.342,2.301) | <0.001 | 1.436 (1.068,1.930) | 0.016 | 1.304 (0.965,1.761) | 0.084 |
| *P* for trend |  | 0.138 |  | 0.074 |  | 0.243 |
| SDH |  |  |  |  |  |  |
| WWI | 1.532 (1.433,1.638) | <0.001 | 1.358 (1.244,1.482) | <0.001 | 1.336 (1.222,1.461) | <0.001 |
| Q1 | 1.000 (Ref) | - | 1.000 (Ref) | - | 1.000(Ref) | - |
| Q2 | 2.918 (2.355,3.617) | <0.001 | 1.807 (1.444,2.262) | <0.001 | 1.734 (1.382,2.730) | <0.001 |
| Q3 | 4.741 (3.848,5.839) | <0.001 | 2.384 (1.909,2.977) | <0.001 | 2.242 (1.790,2.809) | <0.001 |
| Q4 | 5.008 (4.063,6.174) | <0.001 | 2.272 (1.803,2.865) | <0.001 | 2.156 (1.702,2.730) | <0.001 |
| *P* for trend |  | <0.001 |  | <0.001 |  | <0.001 |
| Abbreviations: WWI, Weight-Adjusted Waist Index; HTN, hypertension; ISH, isolated systolic HTN; IDH, isolated diastolic HTN; SDH, systolic-diastolic HTN; CI, confidence interval; OR, odds ratio.  Model 1^a^: Unadjusted.  Model 2^b^: Adjusted for gender, age, ethnicity, employment, education, residence and annual income.  Model 3^c^: Further adjusted for physical activity, sleep time, smoking, alcohol consumption, GLU, HDL-C, LDL-C, and family history of cardiovascular disease. | | | | | | |

| Table S5. Sensitivity analysis: Association between WWI quartiles and hypertension subtypes after further adjustment for BMI in Model 3 | | | | | | |  |
| --- | --- | --- | --- | --- | --- | --- | --- |
| WWI | Model 1^a^ | | Model 2^b^ | | Model 3^c^ | | |
|  | OR (95%CI) | *P* | OR (95%CI) | *P* | OR (95%CI) | *P* | |
| HTN |  |  |  |  |  |  | |
| WWI | 1.757 (1.646,2.876) | <0.001 | 1.351 (1.248,1.462) | <0.001 | 1.192 (1.095,1.297) | <0.001 | |
| Q1 | 1.000 (Ref) | - | 1.000 (Ref) | - | 1.000 (Ref) | - | |
| Q2 | 2.225 (1.853,2.673) | <0.001 | 1.487 (1.223,1.808) | <0.001 | 1.256 (1.024,1.539) | 0.028 | |
| Q3 | 3.820 (3.204,4.556) | <0.001 | 2.158 (1.782,2.612) | <0.001 | 1.611 (1.318,1.969) | <0.001 | |
| Q4 | 4.247 (3.565,5.060) | <0.001 | 2.114 (1.732,2.580) | <0.001 | 1.490 (1.207,1.840) | <0.001 | |
| *P* for trend |  | <0.001 |  | <0.001 |  | <0.001 | |
| ISH |  |  |  |  |  |  | |
| WWI | 2.476 (2.214,2.769) | <0.001 | 1.454 (1.276,1.658) | <0.001 | 1.343 (1.174,1.536) | <0.001 | |
| Q1 | 1.000 (Ref) | - | 1.000 (Ref) | - | 1.000 (Ref) | - | |
| Q2 | 1.901 (1.299,2.784) | 0.001 | 1.382 (0.922,2.072) | 0.117 | 1.242 (0.824,1.872) | 0.300 | |
| Q3 | 3.759 (2.643,5.347) | <0.001 | 1.994 (1.358,2.927) | <0.001 | 1.648 (1.116,2.433) | 0.012 | |
| Q4 | 7.362 (5.287,10.253) | <0.001 | 2.453 (1.683,3.578) | <0.001 | 1.920 (1.370,2.821) | 0.001 | |
| *P* for trend |  | <0.001 |  | <0.001 |  | <0.001 | |
| IDH |  |  |  |  |  |  | |
| WWI | 1.298 (1.166,1.445) | <0.001 | 1.181 (1.037,1.346) | 0.012 | 1.027 (0.895,1.178) | 0.705 | |
| Q1 | 1.000 (Ref) | - | 1.000 (Ref) | - | 1.000 (Ref) | - | |
| Q2 | 1.818 (1.377,2.401) | <0.001 | 1.322 (0.991,1.765) | 0.058 | 1.100 (0.819,1.476) | 0.527 | |
| Q3 | 2.836 (2.174,3.701) | <0.001 | 1.953 (1.472,2.591) | <0.001 | 1.453 (1.087,1.944) | 0.012 | |
| Q4 | 1.782 (1.333,2.383) | <0.001 | 1.372 (0.998,1.885) | 0.051 | 0.982 (0.707,1.365) | 0.916 | |
| *P* for trend |  | 0.015 |  | 0.134 |  | 0.742 | |
| SDH |  |  |  |  |  |  | |
| WWI | 1.764 (1.615,1.926) | <0.001 | 1.377 (1.236,1.534) | <0.001 | 1.194 (1.064,1.340) | 0.003 | |
| Q1 | 1.000 (Ref) | -- | 1.000 (Ref) | - | 1.000 (Ref) | - | |
| Q2 | 2.888 (2.189,3.811) | <0.001 | 1.779 (1.335,2.371) | <0.001 | 1.465 (1.090,1.970) | 0.011 | |
| Q3 | 4.998 (3.831,6.520) | <0.001 | 2.606 (1.972,3.443) | <0.001 | 1.879 (1.408,2.507) | <0.001 | |
| Q4 | 5.279 (4.047,6.886) | <0.001 | 1.779 (1.335,2.371) | <0.001 | 1.745 (1.293,2.356) | <0.001 | |
| *P* for trend |  | <0.001 |  | <0.001 |  | <0.001 | |
| Abbreviations: WWI, Weight-Adjusted Waist Index; HTN, hypertension; ISH, isolated systolic HTN; IDH, isolated diastolic HTN; SDH, systolic-diastolic HTN; CI, confidence interval; OR, odds ratio.  Model 1^a^: Unadjusted.  Model 2^b^: Adjusted for gender, age, ethnicity, employment, education, residence and annual income.  Model 3^c^: Further adjusted for physical activity, sleep time, smoking, alcohol consumption, BMI, GLU, HDL-C, LDL-C, and family history of cardiovascular disease. | | | | | | | |

| Table S6. Multicollinearity diagnostics for covariates in the primary Model 3 | | | |
| --- | --- | --- | --- |
| Variables | GVIF | Df | GVIF^(1/(2*Df)) |
| WWI | 1.28073 | 1 | 1.13169 |
| gender | 1.37379 | 1 | 1.17209 |
| age | 2.25160 | 1 | 1.50053 |
| ethnicity | 1.03263 | 1 | 1.01618 |
| employment | 2.49399 | 3 | 1.16453 |
| physical activity | 1.21131 | 1 | 1.10060 |
| smoking | 1.28416 | 1 | 1.13321 |
| alcohol consumption | 1.29240 | 1 | 1.13684 |
| GLU | 1.02718 | 1 | 1.01350 |
| family history of CVD | 1.02587 | 1 | 1.01285 |
| sleep time | 1.17692 | 1 | 1.08486 |
| residence | 1.34518 | 1 | 1.15982 |
| education | 2.01265 | 4 | 1.09137 |
| annual income | 1.30183 | 2 | 1.06817 |

| Table S7. Multicollinearity diagnostics for covariates in the sensitivity analysis | | | |
| --- | --- | --- | --- |
| Variables | GVIF | Df | GVIF^(1/(2*Df)) |
| WWI | 1.30627 | 1 | 1.14292 |
| gender | 1.37638 | 1 | 1.17319 |
| age | 2.37972 | 1 | 1.54263 |
| ethnicity | 1.03157 | 1 | 1.01566 |
| employment | 2.53509 | 3 | 1.16770 |
| physical activity | 1.20534 | 1 | 1.09788 |
| smoking | 1.28195 | 1 | 1.13223 |
| alcohol consumption | 1.29130 | 1 | 1.13636 |
| GLU | 1.03495 | 1 | 1.01732 |
| family history of CVD | 1.02657 | 1 | 1.01320 |
| sleep time | 1.17723 | 1 | 1.08500 |
| residence | 1.34614 | 1 | 1.16023 |
| education | 1.99619 | 4 | 1.09025 |
| annual income | 1.29665 | 2 | 1.06710 |
| BMI | 1.10169 | 2 | 1.04962 |


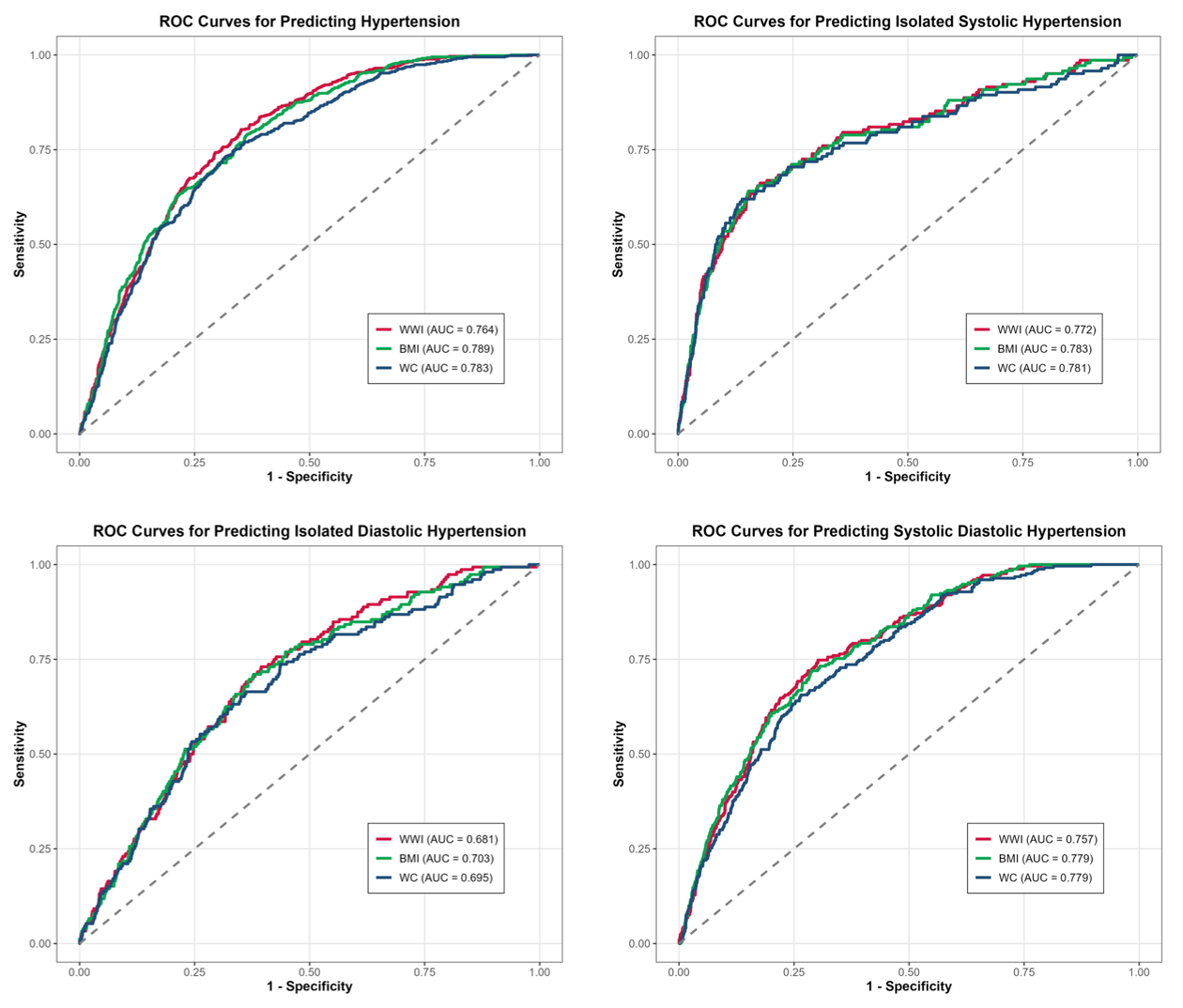


Fig. S1.ROC curves of WWI, BMI, and WC for predicting hypertension and its subtypes.
